# Colandr

**DOI:** 10.5195/jmla.2021.1263

**Published:** 2021-07-01

**Authors:** Melissa Kahili-Heede, K. J. Hillgren

**Affiliations:** 1 mkahili@hawaii.edu, Information Services and Instruction Librarian, Health Sciences Library, University of Hawaii, Manoa, Honolulu, HI; 2 hillgren@hawaii.edu, Health Sciences Library, University of Hawaii, Manoa, Honolulu, HI

## Abstract

**Colandr.**https://colandrcommunity.com;colandrteam@gmail.com; pricing: free.

## INTRODUCTION

The systematic review process can be long and complicated and should include a team of reviewers to reach the proper conclusions [1]. At the most basic level, a systematic review entails formulating a research question, developing a protocol, constructing a search strategy, executing the search across multiple databases, screening titles and abstracts for inclusion and exclusion based on the protocol, reviewing full-text articles, and then comparing and analyzing the data to draw conclusions [1]. There are a number of different systematic review software tools available for researchers to choose from. Few are designed to aid in the entire process, which often requires researchers to deploy several different tools for each of the steps required. The few notable tools that do address multiple steps are Covidence, DistillerSR, and JBI SUMARI [2]. Others target one or two steps. Rayyan, for example, helps facilitate title, abstract, and full-text screening, but has no function for data extraction. With so many options available, it can be difficult for researchers and librarians to know which tools to use.

## ABOUT COLANDR

Colandr is a free, open-source, web-based, research and evidence synthesis tool that operates using machine learning. It is designed to facilitate collaboration throughout various steps of the systematic review process. We were first introduced to Colandr in 2017, but the early version was glitchy to work with. A few review articles from its early days comment on difficulties but note its potential promise compared to a manual process [3, 4]. We opted to use Rayyan but ultimately found ourselves wishing for a data extraction function. Not wanting to pay for yet another tool, we decided to give Colandr a try again in 2020, and overall it shows considerable improvement from the early days of its implementation.

**Figure 1:**
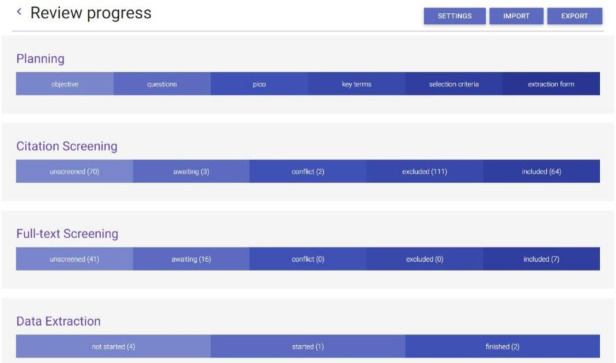
Colandr review workflow

The Colandr interface is divided into four sections that reflect the various stages of a systematic review: Planning, Citation Screening, Full-text Screening, and Data Extraction (Figure 1). This resource review will outline the use of Colandr from the beginning to the end of a systematic review. We piloted the Colandr interface using a scoping review research question related to health literacy.

## THE COLANDR WORKFLOW

### Create a review

The first step when using Colandr is to create a systematic review project. Colandr allows the user to control who owns the review. For example, the person who initially sets up the review is by default the “owner” of the review. There is an option to add other collaborators and to designate one of them as the owner. This is beneficial for librarians who may be assisting researchers on systematic reviews. The owner of the review can add other collaborators and controls the review settings, such as designating whether the review requires one or two reviewers per citation. The owner is also the only one who can edit the protocol created in the next stage, Planning.

### Planning

The Planning stage entails defining the review objectives and research questions using the PICO (patient, population, or problem; intervention; comparison; outcome) model to create a clinical framework, identifying key search terms and selection criteria, and creating an extraction form. The Planning stage details can be seen by all reviewers but can only be edited by the designated owner of the review. It is important to note that the ability to use filters, screens, and ranks in the next step, Citation Screening, is contingent on the Planning workspace being adequately filled out. The organization of the Planning workspace in Colandr is helpful not only for reviewers to ensure they are addressing the necessary steps in the review process, but it also serves as a reference for reviewers and helps to standardize the process.

### Citation Screening/Import

The next step is Citation Screening. At this stage of the workflow, users are ready to upload the results from the database searches. The preferred Colandr file type is RIS, but the web application should also be able to upload TXT and BIB files. We recommend exporting search results from each respective database, importing them into a citation manager such as Zotero, removing duplicates, exporting from Zotero in RIS format, then importing that RIS file into Colandr. Colandr does have an automatic deduplication process, but it only works on citations that are precisely the same. Users will notice that after uploading citations, the authors of articles will appear listed in alphabetical order. Colandr states that this happens to aid in their deduplication process. Also, it is worth noting that once citations are uploaded to Colandr, there is no function to delete them, so it is best to upload them carefully.

### Full-text Screening

Full-text screening capability is one of the features that sets Colandr apart from other tools. At this stage of the process, full-text PDFs are uploaded to Colandr for review. The benefit is that the machine learning algorithm can assist in the process once some of the articles have been reviewed and marked for inclusion or exclusion. The Colandr system will learn which combinations of words and phrases are more relevant to the user [3]. One of the drawbacks, however, is that there is no batch upload option for PDFs. It is a manual process to be completed one article at a time. Fortunately, all collaborators can assist with uploading PDFs, not just the owner of the review. As the full text is reviewed, each individual reviewer can decide whether to include or exclude an article. If the article is marked for inclusion, it will move on to the final stage, Data Extraction.

### Data Extraction/Export

The data extraction form fields are set in the Planning phase of the review process but can be edited as one moves through the review process. Users may decide to revisit or set up the data extraction form after completing some of the initial screening. It is important to note that each data extraction field must be saved before editing the following field. As data extraction fields are created in the Planning section, there is the option to set the data type or value (e.g., integer, text, etc.). This function allows standardization of the data collected among multiple reviewers.

**Figure 2:**
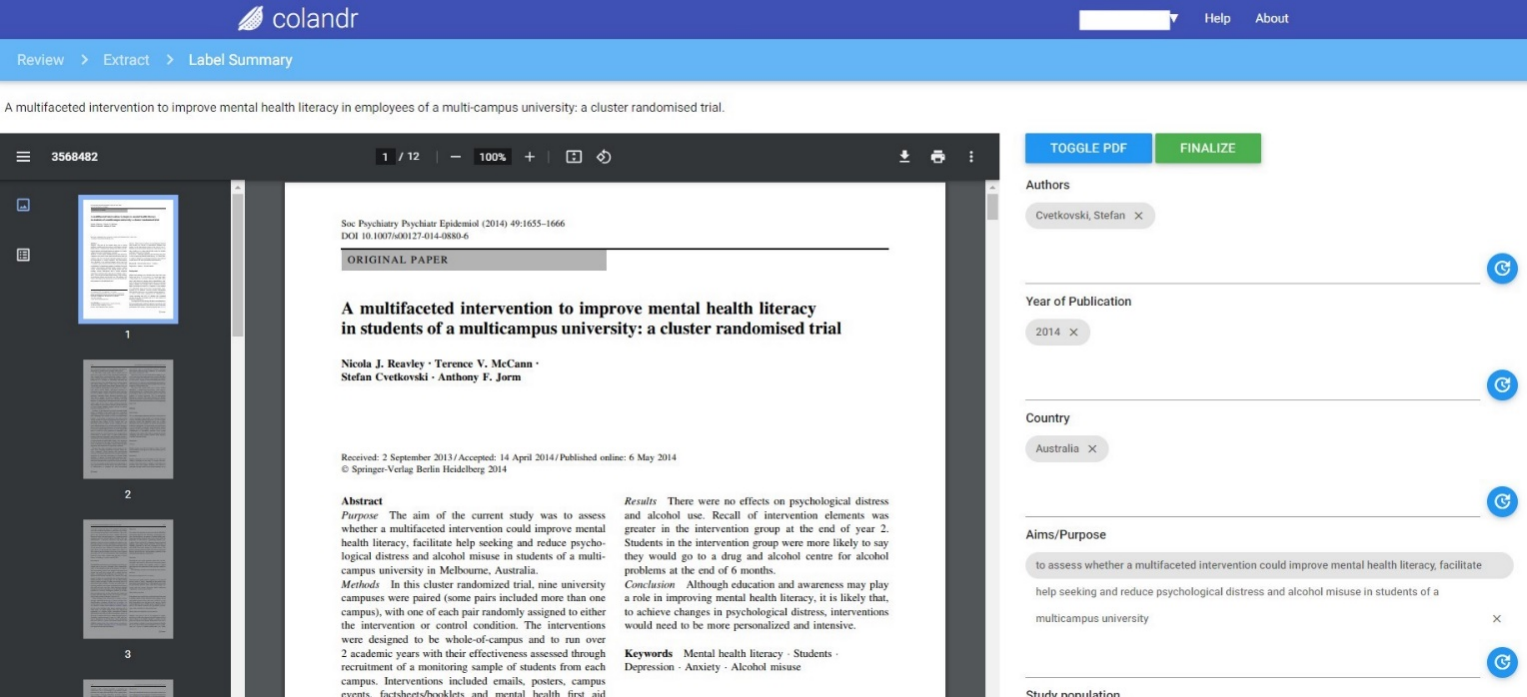
Data extraction form with PDF view

The data extraction function can be clunky, requiring the user to reload the page to ensure what has been entered is saved. We noticed that brief data items (e.g., n=13) being extracted were saved more seamlessly than longer data (e.g., uncontrolled single-group design along with pre-post and 6-week follow-up questionnaire and qualitative interviews 1–2 weeks post-intervention) (Figure 2). However, we find the Colandr data extraction option still has the advantage over a manual process.

## CONCLUSION

A recent study by Harrison et al. assessed systematic review tools to support the title and abstract phase of the systematic review process and ranked Rayyan and Covidence as the most favorable tools compared to others reviewed [5]. We want to add Colandr to the list of most favorable tools. Like Rayyan, a systematic review tool that many may already be familiar with, Colandr utilizes similar machine learning processes. Unlike Rayyan, Colandr has an automatic deduplication process that can help reduce some of the workload by automatically deleting exact duplicate records. In contrast, Rayyan needs the user to review duplicate records before they are deleted. Colandr is similar to Covidence in terms of its data extraction feature, but unlike Covidence, it is a free and open-source tool. One other advantage is the way the Colandr system allows for multiple users to work on the same project. In our trial, one librarian focused on locating and adding full text while the owner librarian could tag and include articles at multiple stages. Collaborators' ability to work on different stages of the review simultaneously allows reviewers with other skills to work independently while still moving the project forward.

Overall, Colandr can be especially useful for librarians working with researchers on systematic reviews. Librarians could use this tool to help set up the review process for researchers by assisting in the Planning stage and citation upload. We know the importance of including librarians in systematic reviews, and Colandr is a tool that librarians and researchers can quickly learn and implement in their projects. Systematic reviews that include librarians are “much higher quality, both in terms of the search strategy itself and search strategy reporting” [6]. This is likely because systematic reviews play to our strengths, rather than to those of researchers. Medical researchers and specialists are not typically conversant in complex search processes and multiple databases [7]. In addition to making a review more comprehensive, librarians are more likely to include multiple databases and gray literature to reduce bias [8].

Despite the minor difficulties experienced while piloting Colandr, we find the tool to be highly useful. As a free and open-source tool, it shows incredible promise. It is easy and affordable for researchers to adopt, requiring only an email address to sign up. As an open-source tool, it is reliant on volunteers to maintain and improve the application. Similar to the open-source citation management tool Zotero, which also relies on volunteers to improve it, we think Colandr will continue to improve as more and more users engage with it.
